# Planar Gas Expansion under Intensive Nanosecond Laser Evaporation into Vacuum as Applied to Time-of-Flight Analysis

**DOI:** 10.3390/e24121738

**Published:** 2022-11-28

**Authors:** Alexey Morozov, Vladimir Titarev

**Affiliations:** 1 Kutateladze Institute of Thermophysics of the Siberian Branch of the Russian Academy of Sciences, Lavrentyev Ave. 1, Novosibirsk 630090, Russia; 2 Federal Research Center “Computer Science and Control” of the Russian Academy of Sciences, Vavilova Str. 44/2, Moscow 119333, Russia

**Keywords:** DSMC, BGK model, gas expansion, pulsed laser evaporation, time-of-flight, rarefied gas, Nesvetay, LasInEx, discrete velocity scheme, ALE

## Abstract

A computational investigation of the dynamics of gas expansion due to intense nanosecond laser evaporation into vacuum has been carried out. The problem is solved in a one-dimensional approximation, which simplifies calculations and at the same time allows one to analyze the main features of the expansion dynamics. For analysis we use three different approaches. Two of them are based on kinetic analysis via the direct simulation Monte Carlo (DSMC) method and numerical solution of the model Bhatnagar–Gross–Krook (BGK) equation. The third one focuses on derivation of an analytical continuum solution. Emphasis is placed on the analysis of the velocity distribution function and the average energy of particles passing through the time-of-flight detector on the normal to the evaporation surface, which is important for interpreting experimental measurements. The formulated problem is quite difficult as the considered flow is time-dependent, contains discontinuities in boundary conditions and involves large variations of local Knudsen numbers as well as steep gradients of the velocity distribution function. Data were obtained on the particle energy in the time-of-flight distribution for the range of regimes from the free molecular flow to continuum one. The maximum attainable average energy of particles in the time-of-flight distribution is determined. The non-monotonicity of the energy increase was found, which is explained based on analysis of the velocity distribution of particles.

## 1. Introduction

Various modern technologies for thin film deposition, nanoparticle synthesis, and surface treatment employ pulsed laser ablation of solid targets with nanosecond pulses of moderate intensity [1]. Such a process leads to the formation of a vapor cloud of the ablation products, which then expands into the surrounding space. Investigation of the dynamics of this process is useful in applications to control and monitor the gas phase.

In experiments, a small detector is usually located at a large distance from the target in the direction normal to the evaporation surface. One of the main instruments to experimentally control the laser ablation and desorption processes is the measurements of the so-called time-of-flight (TOF) distributions of particles passing through this detector [2,3,4,5,6,7,8,9]. By analyzing the TOF distributions, one can improve understanding of the ablation mechanism as well as estimate the temperature of the evaporating surface [6] and the composition of the surface material [10]. Correct interpretation of the TOF distributions can significantly advance the understanding of the processes accompanying pulsed laser ablation and hence facilitate the development of various laser ablation-based techniques.

The theoretical analysis of the problem in question is based mostly on various computational approaches. For low laser fluence the gas can be considered neutral since the effects of laser radiation absorption in the plume and gas ionization are negligible. Typically, the neutral plume expansion in vacuum is studied numerically using the direct simulation Monte Carlo (DSMC) method [11]. In the first works this method made it possible to study angular distributions of particles under pulsed desorption of a few monolayers based on one-dimensional calculations [12,13]. Later, based on two-dimensional calculations, the influence of the size of the evaporation spot on the expansion of particles was investigated [14] and the structure of the forming laser-induced plume and its expansion dynamics has been studied [15]. The influence of chemical reactions [16] and the interatomic interaction potential in the plume [17] on the expansion dynamics has been investigated as well. The effect of the pressure of the evaporated substance on the forming angular distributions of particles has been studied [18]. Special attention was paid to the effects of separation of the components of the gas mixture during ablation of multicomponent substances [10,19,20].

In a number of papers, the TOF distributions under pulsed laser evaporation into vacuums have been analyzed. The influence of the heat of vaporization and chemical reactions on the TOF distribution was studied [21]. Introduction of high-energy particles into the calculation to take into account the effect of ion recombination made it possible to describe TOF distributions under the conditions of plasma formation in the laser plume [22]. It was shown that using the calculated database of TOF distributions allows determining the irradiated surface temperature from the experimental TOF signals [23], while the commonly used fitting formulas greatly overestimate the surface temperature [24]. It was explained why the energy of particles in the TOF distribution can be several times higher than the energy of particles during evaporation and it was shown how this energy depends on the number of evaporated monolayers Θ [25,26]. It was shown that taking into account the time evolution of laser irradiation [27,28] and its spatial non-uniformity [29] has little effect on the energy and the velocity distribution function (VDF) of particles at the TOF detector.

However, the obtained data on the dependence of energy in the TOF distribution on the number of evaporated monolayers Θ were not completely clear and explainable. The previous calculations show a complicated dependence of the energy on the number of monolayers Θ with the formation of a bend [25,26] or even a flat-shaped region for large evaporation spots [28,29] for 1 < Θ < 10, followed by a further increase in energy at 10 < Θ < 100. This contradicts the general idea that, in the limit, the energy should approach to a certain value corresponding to the continuum solution.

Such a strange behavior of the energy dependence gave reason to assume that the calculations for Θ > 10 were inaccurate. It would be interesting and useful to determine the maximum achievable energy in the continuum limit. However, it is very difficult to solve this problem in the axisymmetric formulation under intense evaporation (for Θ > 100, when we approach the continuous regime), since this requires large computing resources. On the other hand, it is possible to derive an exact solution in the one-dimensional formulation and, on its basis, analyze the flow in the entire range of rarefaction, up to the continuous medium.

For nanosecond laser ablation, the radius of the spot is usually significantly larger than the plume length during the pulse action. As a result, the initial stage of the plume expansion can be considered as one-dimensional, which paves the way to commonly used simplified theoretical analysis [12,13,17,30,31,32]. This is vital because in laser ablation applications only a small amount of material typically evaporates and therefore the molecular collisions inside the plume occur only during the one-dimensional expansion. The subsequent three-dimensional expansion can be considered collisionless, greatly simplifying the analysis. Normally, the collisional stage of the gas expansion ends at a distance similar in size to the evaporation spot (~0.1 mm). Since the distance to the detector is much greater (~100 mm), the collisionless expansion can be regarded as if from a point source. Therefore, to estimate the energy of the particles on the TOF detector, it is enough to compute the energy of those particles that move along the normal to the surface inside a cone with a small opening angle. An analysis of the time evolution of this energy makes it possible to trace the transition to the collisionless stage of the gas expansion and to determine the energy of particles arriving at the TOF detector. The initial stage of the time evolution of the energy for a given duration of evaporation will be the same for both one-dimensional and three-dimensional calculations. The larger the evaporation spot is, the longer the three-dimensional calculation corresponds to the one-dimensional one.

Numerically, the formulated problem is quite difficult as the considered flow is time-dependent, contains discontinuities in boundary conditions and involves large variations of local Knudsen numbers as well as steep gradients of the VDF. To make our results more reliable and credible, in addition to the DSMC method, it is worth adding another modeling approach—the direct numerical solution of the model Bhatnagar–Gross–Krook (BGK) kinetic equation computed by the Nesvetay code [33,34]. Previously, good agreement has been obtained using this code both with the DSMC method and with the numerical solution of the exact Boltzmann equation for moderate evaporation (for the number of monolayers Θ < 100) [33]. However, the question of solving the problem under conditions of nearly continuum regime remains open. Traditional discrete velocity methods (DVM) for kinetic equations are highly inefficient when applied to the problem under consideration. The recent incorporation [35] of the Arbitrary Lagrangian-Eulerian (ALE) methodology combined with the introduction of the special unstructured mixed-element velocity meshes [33] allows us now to compute the solution of the problem up to 100 times faster and hence has made it possible to significantly improve the accuracy of calculations and analyze the particle velocity distribution in the laser plume.

The present work focuses on the particular case of the plane expansion into vacuum during intense evaporation. The emphasis is made on the analysis of the VDF and the average energy of particles passing through the time-of-flight detector normal to the evaporation surface, which is important for interpreting experimental measurements. In our analysis we employ three methods. First, two methods are kinetic—the DSMC method and the numerical solution of the model BGK equation. The third method, which is only possible in the planar expansion case, is the analytical continuum solution, which approaches the kinetic solutions for the number of monolayers greater than 1000. The use of three independent analysis methods makes it possible to cross verify the results and increase the credibility of the analysis. The paper also demonstrates the good potential of using model kinetic equations to study flow problems, which need an accurate calculation of not just mean quantities (density, velocity, etc.), but also of the velocity distribution function.

## 2. Formulation of the Problem

A one-dimensional planar problem of pulsed evaporation of molecules into vacuum is considered. The laser-induced plume is assumed to be neutral. The mechanism of normal evaporation [36] is supposed when the relation between the surface temperature and the saturated gas pressure is described by the Clausius-Clapeyron equation. This mechanism is commonly considered to be adequate for describing experiments for moderate laser fluences for nanosecond ablation of different materials, e.g., metals, semiconductors, or graphite [37].

Molecules are evaporated with the energy corresponding to a surface temperature $T_{0}$. It is assumed that during time interval τ particle flux Ψ is constant and equal to $\Psi =n_{0}u_{T}/4$, where $n_{0}$ is the density of the saturated vapor corresponding to the temperature $T_{0}$, $u_{T}=2u_{0}/\sqrt{\pi }$, $u_{0}=\sqrt{2kT_{0}/m}$ is the most probable thermal speed, *k* is the Boltzmann constant, *m* is the mass of an evaporated molecule. All backscattered molecules which reach the evaporating surface are assumed to recondense on the surface. The monatomic gas is considered. The hard sphere model is used to simulate the process of particle collisions.

The concept of evaporated monolayers is often used [14,16,26] to describe the amount of evaporated material. One monolayer corresponds to such a number of particles that they cover the evaporating surface completely. The total number of evaporated molecules is equal to

(1) $$N_{vap}=\tau \Psi_{vap}S,$$

where *S* = π*R*^2^ is the spot area. The number of evaporated monolayers is defined as

(2) $$\Theta =\frac{N}{S/\Sigma }=\tau \Psi_{vap}\Sigma =\frac{\tau }{8\sqrt{2\pi }t_{0}},$$

where Σ = σ/4 is an area occupied by one molecule at the surface and $t_{0}=\lambda_{0}/u_{0}$ is the average time between collisions in the saturated vapor with density $n_{0}$ and temperature $T_{0}$, $\lambda_{0}$ is the mean free path. Since we consider regimes up to the continuum medium, the interesting range of monolayers is Θ = 0.01–10,000. To characterize the degree of rarefaction, one can determine the Knudsen number at the initial stage of expansion based on the plume length *L* = $u_{T}\tau$ as

(3) $$Kn=\frac{\lambda_{0}}{L}=\frac{1}{16\sqrt{2}\Theta }.$$

The indicated range of the number of monolayers Θ corresponds to the range of Knudsen numbers *Kn* = 4–4 × 10^−6^. It is obviously that it ranges from a continuum solution to a free-molecular one.

Let us estimate the number of model particles required for simulating a typical near-continuum regime by the DSMC method in the axisymmetric formulation. The gas density near the evaporation surface during evaporation is close to density at the boundary of the Knudsen layer $n_{K}$. To correctly simulate the gas flow near the surface, the cell size should be no larger than the local mean free path ∆*x* = $\lambda_{K}$ = $\lambda_{0}n_{0}/n_{K}$. In addition, there must be at least one model particle in the smallest cell in the computational domain (cell size of ∆*x*^3^, near the flow axis). The maximum density and the maximum number of particles in the flow field are realized at the moment of time *t* = τ. At this time, the total number of real molecules in the plume in the continuum limit can be estimated as $N_{mol}=n_{K}u_{K}\tau S$, where $u_{K}$ = $c_{K}$ = $\sqrt{\gamma kT_{K}/m}$ is velocity at the boundary of the Knudsen layer (where the Mach number *M* = *u*/*c* = 1), $T_{K}$ is the temperature at the boundary of the Knudsen layer, γ = (5 + *j*)/(3 + *j*) is the adiabatic exponent, and *j* is the number of internal degrees of freedom.

The total number of *model* particles in one-dimensional plane modeling can be estimated as the ratio of the total number of molecules in the plume to the number of molecules in the smallest cell near the surface:

(4) $$N_{model,1D}=\frac{n_{K}u_{K}\tau \Delta x^{2}}{n_{K}\Delta x^{3}}=\frac{u_{K}\tau }{\lambda_{K}}=\frac{8n_{K}\Theta }{n_{0}}\sqrt{\frac{\gamma \pi T_{K}}{T_{0}}}\approx 4.7\cdot \Theta .$$

Here, we use the data on the values at the Knudsen layer boundary obtained by the numerical solution of the model kinetic equation [38]

(5) $$T_{K}/T_{0}=0.6434,\;n_{K}/n_{0}=0.3225.$$

To estimate the number of model particles in the axisymmetric calculation, it is necessary to compare the evaporation spot area *S* = π*R*^2^ with the area of one cell $S_{1}$ = ∆*x*^2^. Expressing the spot radius in dimensionless form as $b=R/\left(u_{T}\tau \right)$, we obtain

(6) $$\frac{S}{S_{1}}=\frac{\pi b^{2}u_{T}^{2}\tau^{2}}{\Delta x^{2}}=512\pi {\left(\frac{n_{K}}{n_{0}}\right)}^{2}b^{2}\Theta^{2}=167\;b^{2}\Theta^{2}.\;$$

The total number of model particles in the axisymmetric calculation is

(7) $$N_{model,3D}=\frac{S}{S_{1}}N_{model,1D}=792\;b^{2}\Theta^{3}.\;$$

For example, for a typical evaporation spot *b* = 10 and Θ = 1000 we obtain *N_model_*_,3*D*_ ≈ 8 × 10^13^, which is beyond the limits of possible computational possibilities, while for one-dimensional calculation the minimum number of model particles is only *N_model_*_,1*D*_ ≈ 5 × 10^3^. It should be noted that the axisymmetric DSMC calculation requires the same large number of model particles as the corresponding three-dimensional calculation, and only the number of cells in physical space decreases. In principle, the number of model particles can be significantly reduced by using in the radial direction the cell size larger than the mean free path (and thus increasing the volume of the smallest cell) or by using weighting factors. However, such approaches are nontrivial and can also distort the calculation results. Sometimes, the cell size is set larger than the mean free path, which also makes it possible to reduce the number of model particles, but in this case, the accuracy of the resulting numerical solution requires a separate study [39]. Thus, it can be seen that the high-accurate numerical solution of this problem by the DSMC method in the axisymmetric formulation is exceedingly computational costly, if even possible, which explains the need to use the one-dimensional approach.

## 3. Methods of the Analysis

### 3.1. DSMC

The first of the two considered numerical approaches is the DSMC scheme. We use the standard version of the method [11] with some improvements borrowed from [40]. Broadly speaking, the DSMC approach works as follows. The gas cloud is described by the so-called model molecules. The state of each molecule is determined by its position in space and the velocity vector. Temporal evolution of the flow field during one time step is conducted via the so-called time splitting approach and consists of two stages. The first stage is collisionless movement of particles over the mesh in the physical domain, based on their position and velocity. The second stage involves the simulation of the interparticle collisions in accordance with the “no-time-counter” scheme.

The outlined computational DSMC algorithm was implemented by the first author in the parallel FORTRAN code LasInEx (Laser-Induced-Expansion) and has been successfully used in various studies, e.g., [15,20,26,34]. In the current work the computational domain in physical space is initially divided into cells of equal size. Its right boundary is pushed forward so that no particle can escape from the domain. At each moment of time when the domain length is updated and mesh is rebuilt, the maximum density in the computational region was calculated, and the cell size was set equal to the corresponding mean free path. Since the density decreases during the gas cloud plume expansion, the cell size grows accordingly.

### 3.2. BGK Model Equation

The second numerical approach is based on solving numerically the BGK model kinetic equation [41]. The state of the gas at position x at time moment t is described by the velocity distribution function $f\left(t,x,\xi \right)$ where $\xi =\left(\xi_{1},\xi_{2},\xi_{3}\right)$ are the components of the molecular velocity vector. Density, velocity, temperature, and pressure are defined by means of the integrals over the complete velocity space. For the considered one-dimensional problem the kinetic equation reads as follows:

$$\frac{\partial }{\partial t}f+\frac{\partial }{\partial x}\left(\xi_{1}f\right)=\nu \left(f_{M}-f\right),\;\;\nu =\frac{p}{\mu },\;f_{M}=\frac{n}{{\left(2\pi kT/m\right)}^{3/2}}exp\left(-\frac{m{\left(\xi -u\right)}^{2}}{2kT}\right),p=nkT,$$

where ν is the collision frequency. For the hard sphere model, the viscosity coefficient is $\mu \left(T\right)∼\sqrt{T}$.

The standard approach to solve a model kinetic equation for transient problems with sharp gradients is a discrete-velocity method (DVM). The essence of the method consists of replacing the infinite velocity domain by a finite integration domain and subsequently passing from the kinetic equation to the system of equations for finite set of integration points. The resulting system of kinetic equations can be solved by a variety of modern advection schemes [42,43]. However, the traditional DVM schemes are highly inefficient when applied to the problem under consideration. Instead, we use a recent ALE method [35], which uses deforming spatial meshes and expanding spatial domains. For the basic explanation of the ALE approach see, e.g., [44,45]. The test calculations, using the code Nesvetay developed by the second author [46,47,48], showed the ALE-DVM scheme to be up to 100 times more efficient as compared to the conventional DVM methods. It has been recently successfully used for studying gas expansion into background gas [34]. For flow into a vacuum, some modifications of the baseline scheme were made in order to improve its robustness. It is also important to note that to avoid division by zero the initial condition of vacuum is replaced by the background gas with the small number density value 10^−15^ $n_{0}$. Background gas temperature value is not too important and for simplicity is set to be equal to $T_{0}$. Our numerical experiments have shown that the use of even smaller values does not change the outcome of the calculations. The initial value of the VDF is then set to be the locally Maxwellian function with the background gas density and temperature.

In the physical space, the initial domain extends up to 5 cm and is divided into 800 cells. During the calculations, it expands in such a way that the advancing wave never reaches the right boundary of the calculation domain. A specially constructed unstructured mixed-element velocity mesh, proposed in [33], is used in the velocity mesh. This is due the need to calculate the TOF distributions of particles, which involves the integration of the VDF over the cones with a small opening angle in the velocity space. Our approach to velocity mesh construction borrows ideas from computational astrophysics, see [49] and references therein. The mesh topology is different depending on the part of the velocity domain. For $\xi_{1}>0,$ the mesh is constructed by extruding in the radial direction a triangular mesh on a unit sphere. This results in layers of prismatic cells together with one layer of tetrahedrons near the origin. For $\xi_{1}<0,$ we use a conventional hexahedron mesh. Overall, the resulting velocity mesh contains 840 thousand cells and allows the integration for cones with half angles as low as 0.1°.

To reduce the required computing time, the calculations by the Nesvetay code are run on parallel computers using two-level MPI+OpenMP approach. By default, 8 OpenMP threads are assigned to each MPI process.

### 3.3. Analytical Continuum Solution

To obtain a solution in the limiting case of the continuous medium, we use an analytical solution of the continuity and Euler’s equations for pulsed adiabatic expansion of gas desorbing into vacuum [50]. The applicability of this solution to the considered problem is due to the fact that the adiabaticity assumption is violated only in a small subsonic layer near the evaporation surface, while in the rest of the flow field is well satisfied. On the other hand, the continuum description of the flow is violated only at the plume front or for evaporation of a small number of monolayers.

The position of the plume front ${\tilde{x}}_{f}$ and the point of the maximum plume density $x_{max}$ (for *t* > τ) are determined by the relations

(8) $${\tilde{x}}_{f}(\tilde{t})=\frac{\gamma +1}{\gamma -1}\tilde{t},\;{\tilde{x}}_{\max}(\tilde{t})=\frac{\gamma +1}{\gamma -1}\left(\tilde{t}-{\tilde{t}}^{\frac{3-\gamma }{\gamma +1}}\right),$$

where $\tilde{t}$ = *t*/τ, $\tilde{x}=x/\left(u_{K}\tau \right)$. Solution for the zone ${\tilde{x}}_{\max}$< $\tilde{x}$ < ${\tilde{x}}_{f}$ is determined by the formulas for plane continuum unsteady expansion into vacuum [51]

(9) $$\left\{\begin{array}{c} \tilde{u}(\tilde{x},\tilde{t})=1+\frac{2}{\gamma +1}\frac{\tilde{x}}{\tilde{t}},\\ \tilde{c}(\tilde{x},\tilde{t})=1-\frac{\gamma -1}{\gamma +1}\frac{\tilde{x}}{\tilde{t}}, \end{array}\right.$$

where $\tilde{u}=u/u_{K}$, $\tilde{c}=c/c_{K}$, *u* is velocity, *c* is the speed of sound.

Solution in the zone 0 < $\tilde{x}$ < ${\tilde{x}}_{max}$ should be found separately for any particular case of γ [50]. For monatomic gas (γ = 5/3), the solution is

(10) $$\left\{\begin{array}{l} \tilde{t}(\tilde{u},\tilde{c})=\frac{\left(18{\tilde{c}}^{2}+3\tilde{c}\tilde{u}-12\tilde{c}-{\tilde{u}}^{2}+2\tilde{u}+8)(3\tilde{c}+\tilde{u}+2\right)}{108{\tilde{c}}^{3}},\\ \tilde{x}(\tilde{u},\tilde{c})=-\frac{\left(9{\tilde{c}}^{2}-{\tilde{u}}^{2}+4\tilde{u})(3\tilde{c}+\tilde{u}+2)(3\tilde{c}-\tilde{u}-2\right)}{108{\tilde{c}}^{3}}.\;\; \end{array}\right.$$

Further, assuming the adiabatic relation *n* = $\mathrm{const}\cdot c^{2/\left(\gamma -1\right)}$, temperature and density can be calculated. To compare the DSMC calculation with the analytical solution, one should use known values of density and temperature (5) at the boundary of the Knudsen layer.

To calculate the energy of particles moving in a velocity cone with an angle α, an approach based on the Monte Carlo method is used. Based on the analytical solution (9)–(11), we calculate density, velocity, and temperature profiles. Then, at every point in space, the particle velocity components (*u’*, *v’*, *w’*) are generated in accordance with the local Maxwellian VDF and for each particle it is determined whether it is inside the velocity cone with an angle α or not (i.e., whether the conditions *u’* > 0 and $\sqrt{{v^{\prime }}^{2}+{w^{\prime }}^{2}}/u^{\prime }$ < tgα are satisfied). To calculate the average energy, integration is carried out over the entire space, taking into account density at each point.

## 4. Results and Discussion

### 4.1. Distribution of Molecules in the Velocity Cone

**Figure 1:**
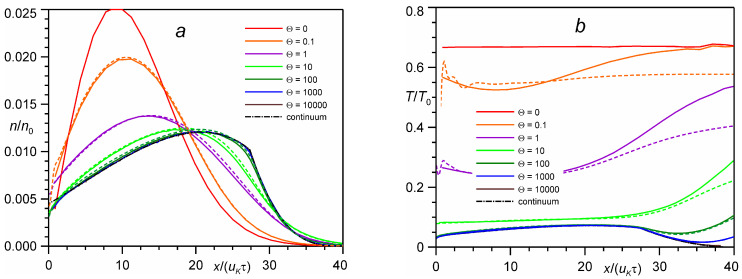
Profiles of density (**a**) and temperature (**b**) in time *t* = 10τ for different numbers of evaporated monolayers Θ: DSMC (*solid lines*) vs. BGK model (*dashed lines*) and continuum solution (*dash-dotted line*).

Figure 1 shows typical density and temperature profiles for various numbers of evaporated monolayers. With an increase in the number of evaporated monolayers, the plume is accelerated with a corresponding drop in temperature due to collisions between particles in the plume. So, for time 10τ, the maximum density value for Θ = 1000 is 2 times smaller as compared to Θ = 0, whereas the maximum temperature is 10 times smaller.

**Figure 2:**
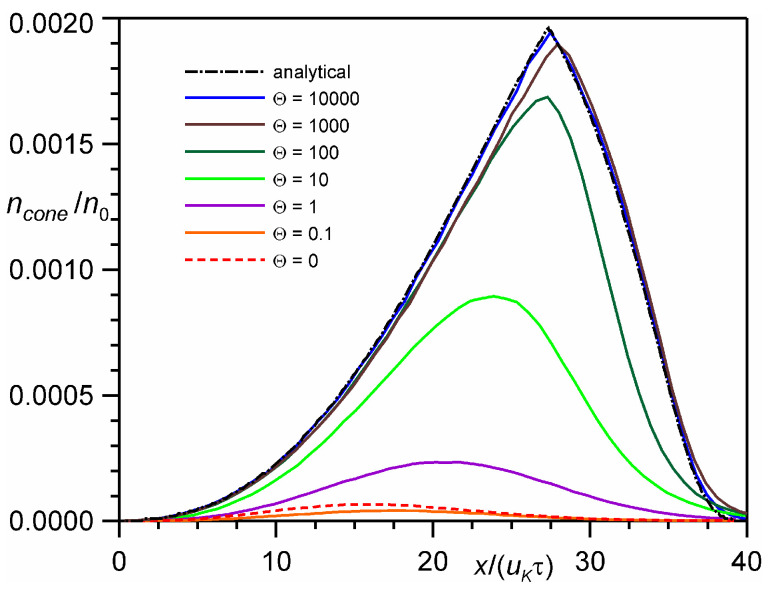
The DSMC calculated spatial distribution of particles moving along the *x* axis in the 3° velocity cone at time *t* = 10τ.

To analyze experimentally measured distributions of particles at a time-of-flight detector, we consider the distribution of particles moving inside a velocity cone with a given angle α. Figure 2 shows numerical results for particles moving along the *x* axis inside a velocity cone with an angle of 3° (i.e., particles for which $\sqrt{{v^{\prime }}^{2}+{w^{\prime }}^{2}}/u^{\prime }$ < tg 3°, where (*u′*, *v′*, *w′*) are the components of the particle velocity vector). It is these particles that will arrive at the time-of-flight detector with a size of *L*tg3° (here *L* is the distance to the detector) under the condition of collisionless expansion. The distribution differs significantly from the one shown in Figure 1a. With an increase in the number of monolayers, the fraction of particles that arrive at the time-of-flight detector increases strongly. It can be seen that faster particles from the plume front with a low temperature arrive at the detector, while slower particles from the plume back with a relatively high temperature move away to the sides. The fraction of particles that arrive at the detector is quite small. One can see that the maximum density of particles in the velocity cone in Figure 2 for Θ ≥ 1000 is 0.002, while the maximum particle density in Figure 1 is 0.012, i.e., 6 times higher. Particles at the plume front (*x* > 30 $u_{K}\tau$) for a large number of monolayers move with almost zero temperature, strictly forward, so most of them will arrive at the detector.

**Figure 3:**
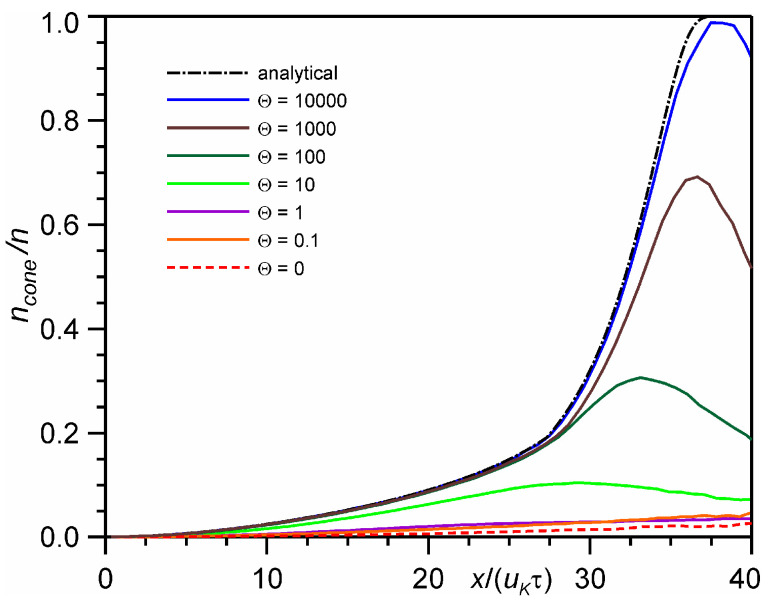
The DSMC calculated probability of a particle to be inside a 3° velocity cone at time *t* = 10 τ.

Figure 3 shows the distribution of the fraction of particles that arrive at the detector with a size of *L*tg3°. In fact, this is equivalent to the probability of a particle being inside the velocity cone with an angle of 3°. It can be seen that with an increase in the number of monolayers from Θ = 10 to 10,000, the probability of a particle arriving at the detector increases from 0.1 to 0.95. For the continuum solution, the probability reaches unity. It is important to note that the numerical solution actually begins to coincide with the analytical continuum solution only for the number of monolayers Θ = 10,000, which corresponds to an extremely small Knudsen number *Kn* = 4 × 10^−6^.

**Figure 4:**
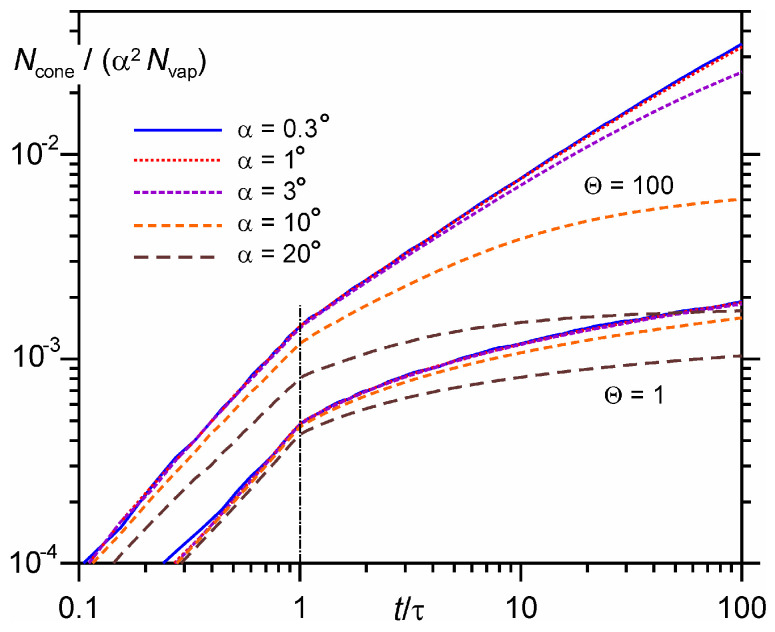
Time evolution of the number of particles moving along the *x* axis in the velocity cone with an angle α = 0.3°, 1°, 3°, 10°, 20° for the number of monolayers Θ = 1 and 100.

The number of particles moving in a velocity cone with an angle α and correspondingly arriving at the detector of size *L*tgα depends on the number of evaporated monolayers Θ and the angle α and varies with time. Figure 4 shows the time evolution of the number of particles for different cone angles. To normalize this number, we use the area of the spherical segment $S^{\prime }\sim 1-\cos\alpha \approx \alpha^{2}/2$. The larger the number of monolayers Θ, the larger the number of collisions between particles during expansion and, correspondingly, the larger the number of particles moving along the normal in the velocity cone. It can be seen that for Θ = 100 for the angle α = 1°, 3% of the evaporated particles arrive at the detector, while for Θ = 1, only 0.2%.

### 4.2. Temporal Evolution of Average Energy of Molecules

**Figure 5:**
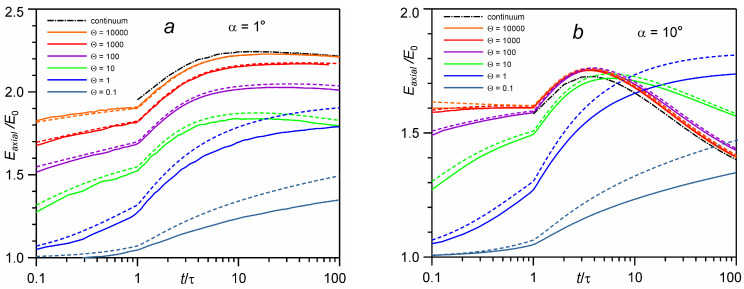
Time evolution of the average energy of particles moving inside a velocity cone with an angle of 1° (**a**) and 10° (**b**) for different numbers of evaporated monolayers Θ.

Figure 5 shows the time evolution of the average energy of particles moving in the velocity cone with different values of the cone angle α. One can see good agreement between the DSMC results and the kinetic equation for Θ > 1. The solution for the number of monolayers 10,000 agrees very well with the analytical continuum solution. For Θ < 1, there is a monotonic increase in energy with time. Similarly, for Θ > 1, the energy increases in the initial period of time, up to *t* = (3–10)τ. This energy rise is caused by two factors, the gas-dynamic acceleration of the plume in the forward direction and the kinetic selection of high-speed particles [26]. In this case, due to separation of molecules for non-stationary expansion, collisions occur mostly between molecules with a close velocity component in the normal direction to the surface. This leads to the transfer of energy from the radial component (along the surface) to the axial component (parallel to the normal to the surface) and to the focusing of molecules in the direction of the normal. This effect has been seen in previous DSMC studies of pulsed evaporation in vacuum [12,14,26]. For Θ ≥ 1, after time *t* = (3–10)τ, the energy begins to decrease. This effect can be explained as follows. With increase in the number of evaporated monolayers, the region of collisional expansion becomes larger. In this case, low-speed molecules, which move to the side at lower values of Θ, undergo additional collisions, which direct them in the forward direction. Thus, the low-speed “tail” of the VDF increases and the total energy decreases accordingly.

**Figure 6:**
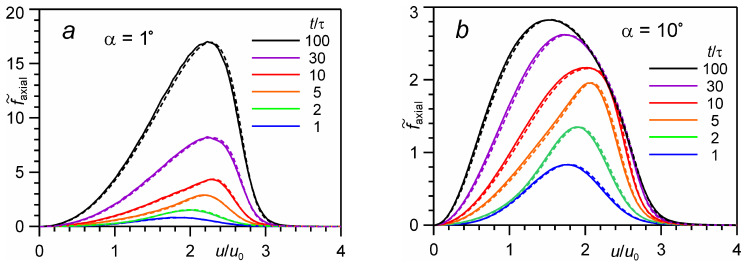
The velocity distribution function of molecules moving along the axis in the cone with angle α = 1° (**a**) and 10° (**b**) for the number of monolayers Θ = 100: DSMC calculation (*solid lines*) in comparison with the BGK model (*dashed lines*).

As an illustration, Figure 6 shows the distributions of those particles that move along the axis *x* inside a cone with an angle of 1° and 10°. The flow regime with strong effect is selected, for Θ = 100. As shown in Figure 4, the number of molecules moving along the axis constantly increases with time. The VDF changes, on the one hand, due to a change in the velocities of molecules moving inside the cone, and on the other hand, due to the appearance of new molecules in the cone. To separate these processes, the function is normalized by the number of molecules in the cone at the end of the evaporation, as ${\tilde{f}}_{axial}\left(t,\alpha ,u\right)=f_{axial}\left(t,\alpha ,u\right)/\int_{0}^{\infty }f_{axial}\left(\tau ,\alpha ,u\right)du$. For *t* = τ this function coincides with the usual VDF.

There is a qualitative difference in the VDF evolution for the small angle (α = 1°) and the large angle (α = 10°). For the small angle initially up to a time of *t* = 10τ there is an increase in the number of high-speed molecules, which leads to an increase in the average energy of these particles. Later, for *t* > 5τ, there is a proportional increase in the number of both fast and slow molecules, which results in the conservation of the average energy of the molecules. However, for the large angle the situation is qualitatively different. After a time of *t* = 5τ, the number of low-speed molecules increases mostly, which considerably decreases the energy *E_axial_*.

It should be noted that the total number of molecules moving inside the cone for α = 1° increases by a factor of 23 during expansion (from 0.0014 at time *t* = τ to 0.033 at time *t* = 100τ, see Figure 4), while for α = 10° only by a factor of 5 (from 0.0012 to 0.006 over the same time interval). It should be expected that with an increase in the number of monolayers (in the continuum limit), with time approaching infinity, all particles should move strictly along the axis. As a result, the energy ratio approaches the unit value $E_{axial}/E_{0}$ = 1.

These data are vital for understanding the patterns observed in two-dimensional calculations. It was found out earlier that for a given number of monolayers with an increase in the size of the evaporation spot (increase in *b* up to 5), the corresponding rise in the energy of particles passing through the TOF detector takes place [25]. However, with further increasing the spot (for *b* > 5), some decreasing energy is observed. Since an increase in the spot size is equivalent to an increase in the duration of the one-dimensional flow regime, the presence of an energy maximum for relatively large spots (*b*~5) is equivalent to the presence of an energy maximum for a relatively long time of one-dimensional calculation (*t*~5τ) and apparently has the same reason. In one-dimensional calculations, it is possible to obtain highly accurate numerical data for almost arbitrarily small angles of the cone and a large number of evaporated monolayers, thus revealing general trends. However, in two-dimensional calculations it is much more difficult due to the requirement of significant computational resources.

### 4.3. Generalizing Dependences on Average Energy of Molecules

**Figure 7:**
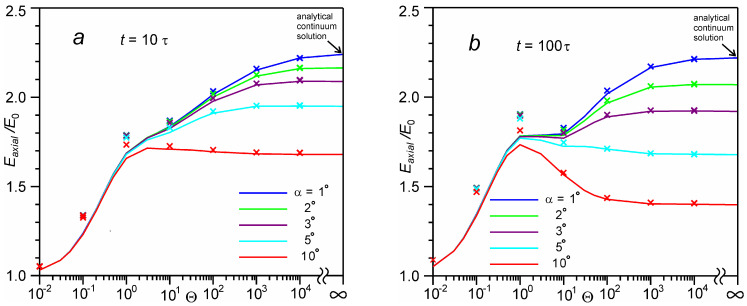
Average axial energy *E_axial_*(*t*, *α*, Θ) as a function of the number of evaporated monolayers Θ at time *t* = 10 τ (**a**) and 100 τ (**b**): DSMC calculation (*solid lines*) in comparison with the BGK model (*crosses*).

Figure 7 depicts average axial energy computed at time *t*/τ = 10 and 100 for different angles. Previously, similar dependences were obtained for *t*/τ = 25 for the number of monolayers Θ < 100 [33]. It can be seen that as the number of monolayers increases from 100 to 10,000, the energy tends to a certain limiting value, which coincides with the analytical continuum solution. At the same time, there is some curve bend in the Section 1 <Θ < 100, and for the angle α ≥ 5°, there is even an energy maximum for Θ = 1. These features are associated with an increase in the number of low-velocity particles in the velocity cone, as shown in Figure 6.

**Figure 8:**
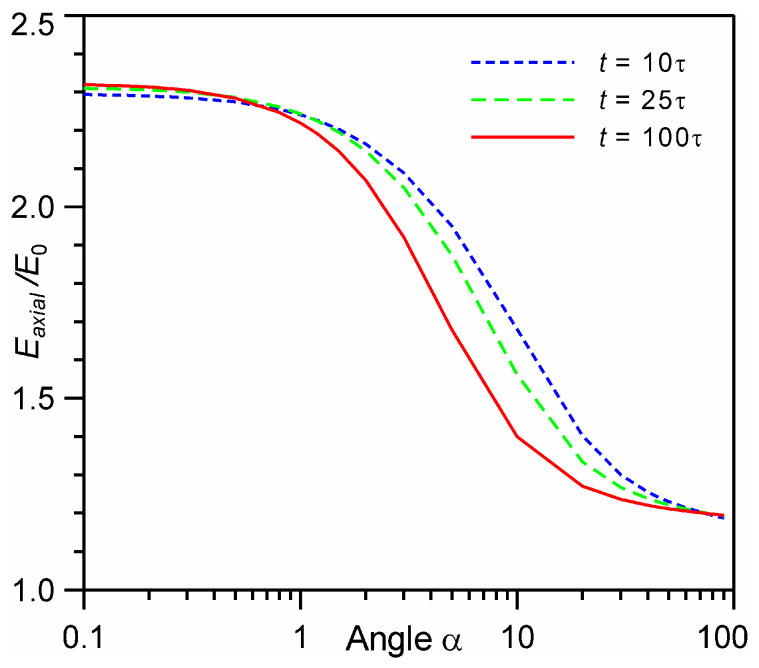
Average axial energy *E_axial_* as a function of the angle α at time *t* = 10, 25, 100τ based on the analytical continuum solution.

The dependences of the average energy of particles in the time-of-flight distribution for *t* = 10τ are similar to those in axisymmetric calculations for a small evaporation spot (*b* = 10) [25,26]. The flat-shaped section of energy for 1 < Θ < 10 for *t* = 100τ in Figure 7b is in good agreement with the flat-shaped section for 1 < Θ < 10 for a large evaporation spot (*b* ≥ 30) [28,29]. It can be concluded that the observed peculiarity of the energy change in the axisymmetric calculation is of the same nature as in our plane calculation. It can also be expected that with an increase of the number of monolayers in the axisymmetric calculation (which is technically difficult due to computational limitations), the energy of particles at the TOF detector should reach some limiting continuum value. This limiting value can be estimated on the basis of our one-dimensional calculations. Figure 8 shows the dependences of the average axial energy on the angle, calculated on the basis of the analytical continuum solution. It can be seen that as the angle decreases, the energy value reaches a certain limit value. Thus, a decrease in the angle from α = 1° to 0.1° leads to an increase in energy by only 4%. It is important to note that the energy value with decreasing angle depends very weakly on time. Increasing the time from 10τ to 100τ only increases the energy by 1%. The maximum achievable energy of particles at the TOF detector is 2.32 $E_{0}$. This corresponds to the case of evaporation from an infinitely large evaporation spot, and in the case of evaporation from any other spot, the energy at the TOF detector should not exceed this value. This is consistent with previous axisymmetric calculations in which the observed energy was less than this maximum value [25,26,28,29].

For the largest possible angle (90°) in Figure 8 the particle energy in the plume is about 1.19*E*_0_. This increase in energy is due to the fact that the total energy of the plume during expansion is not conserved since a considerable fraction of low-energy particles backscatters and recondenses at the evaporation surface [52,53,54]. It was shown that during evaporation of 1000 monolayers, the fraction of particles returning back to the evaporation surface for the entire time of expansion is *β* = 0.275, with the back flux during evaporation being *β*_1_ = 0.163 and after evaporation being *β*_2_ = *β* − *β*_1_ = 0.112 [54]. The corresponding kinetic energies of backscattering particles can be estimated as $E_{\beta ,1}\approx 0.6E_{0}$ [52,53] and $E_{\beta ,2}\approx 0.3E_{0}$ [53]. Then, we can estimate the total plume kinetic energy as *E*_plume_ ≈ $\left(E_{0}-\beta_{1}E_{b,1}-\beta_{2}E_{b,2}\right)/\left(1-\beta \right)\approx 1.2E_{0}$, which agrees well with our analytical estimation.

## 5. Conclusions

The dynamics of gas expansion under intense nanosecond laser evaporation into vacuum is studied. The use of two different kinetic approaches (the DSMC method and the solution of the model BGK equation) allowed obtaining a reliable solution that agrees well with the analytical continuum solution for a large number of monolayers. An analysis of the velocity distribution function and the average energy of particles passing through a time-of-flight detector on the normal to the evaporation surface is carried out. The use of the analytical continuum model made it possible to determine the maximum possible particle energy in the time-of-flight distribution. Based on the analysis of the distribution of particle velocities, the peculiarities of the energy increase in the time-of-flight distribution are explained. The data obtained are important for the interpretation of experimental time-of-flight measurements.
